# Response of Soil Fungal Community to Drought-Resistant *Ea-DREB2B* Transgenic Sugarcane

**DOI:** 10.3389/fmicb.2020.562775

**Published:** 2020-09-18

**Authors:** Xiaowen Zhao, Qi Liu, Sasa Xie, Yuke Jiang, Huichun Yang, Ziting Wang, Muqing Zhang

**Affiliations:** ^1^Guangxi Key Laboratory of Sugarcane Biology, Nanning, China; ^2^State Key Laboratory for Conservation & Utilization of Subtropical Agro-Bioresources, Guangxi University, Nanning, China; ^3^College of Agronomy, Guangxi University, Nanning, China

**Keywords:** drought resistance, *Ea-DREB2B*, transgenic sugarcane, fungal community, environmental factor

## Abstract

Drought limits crop productivity, especially of sugarcane, which is predominantly grown in the subtropical parts of China. Soil microbes perform a wide range of functions that are important for plant productivity and responses to drought stress, and fungi play an important role in plant–soil interactions. The *Ea-DREB2B* gene of sugarcane, *Saccharum arundinaceum*, is involved in regulating the response to drought stress. In this study, fungal communities of the transgenic (TG) sugarcane variety GN18, harboring the drought-tolerant gene *Ea-DREB2B* and its corresponding non-TG wild-type (WT) variety, FN95-1702, were investigated in three soil compartments (rhizoplane, rhizosphere, and bulk soil) by assessing the internal transcribed spacer region using Illumina MiSeq. As the soil microbial community is also affected by various environmental factors, such as pH, carbon availability, and soil moisture, we determined the total carbon (TC), total nitrogen (TN), and total phosphorus (TP) contents in the rhizoplane, rhizosphere, and bulk soil compartments to explore the associations between soil fungal communities and host plant characteristics. The differences between the soil fungal communities of TG and WT plants were detected. The alpha diversity of TG fungal communities was more correlated to environmental factors than the beta diversity. The abundance of operational taxonomic units (OTUs) enriched in TG root-related area was far more than that in the root-related area of WT plants. Thereinto, more saprotrophs were enriched in the TG root-related area, indicating altered niches of fungal guilds around TG roots. These results revealed that host plant genotype did play a key role for strengthening plant–fungi interaction and enhancing beneficial fungal function in the root-related area (rhizoplane and rhizosphere) of TG sugarcane in order to respond to drought stress.

## Introduction

Drought is a major constraint for plant growth and agricultural productivity in many parts of the world. There are several aspects of the plant response to drought, such as sensing stress, activating systemic signaling pathways, and genetically regulating the responses (Zolla et al., 2013). Many genes involved in plant responses to drought have been identified, and some of these have been effectively used to improve drought tolerance (Gosti et al., 1995; Iuchi et al., 2001; Li et al., 2017) by developing new drought-tolerant crop varieties through genetic modifications to increase crop productivity and reduce costs (Romão-Dumaresq et al., 2016).

Soil microbes have a wide range of functions that are important for plant productivity, such as cycling nutrients, inducing disease resistance, and responding to environmental stresses, including drought and salinity (Zolla et al., 2013). Soil fungi play important roles in ecosystem nutrient cycling and as mutualists and pathogens of host plants (Yang et al., 2017). The fungi colonized in endosphere, rhizoplane, and rhizosphere of plant have particularly important feedback effects on the responses of their host plants to climate change (Gehring et al., 2017). Several studies have documented variations in fungal communities among different plant genotypes (Dunfield and Germida, 2004; Hunter et al., 2015). Sugarcane, as a significant resource of sugar and ethanol, has high requirements for irrigation and is very sensitive to water shortage (Ferreira et al., 2017); therefore, genetic engineering has been applied to enhance its drought resistance (Ramiro et al., 2016).

The genes encoding dehydration-responsive element-binding (DREB) transcription factors recognized in *Arabidopsis thaliana* have been reported to enhance drought tolerance in genetically modified plants (Mizoi et al., 2012). *Ea-DREB2B* is a member of the DREB family and cloned from the hardy sugarcane *Saccharum arundinaceum*. It regulates the expression of a few stress-inducible genes and plays a crucial role in promoting plant resistance to drought and salinity (Lata and Prasad, 2011; Augustine et al., 2015). The transgenic (TG) sugarcane (GN18) harboring *Ea-DREB2B* was obtained by genetic transformation using a gene gun using inducible promoter RD29A and sugarcane variety FN95-1702 as the receptor material. Xu et al. (2018) reported that TG sugarcane (GN18) has significantly better drought resistance than non-TG sugarcane. Abscisic acid (ABA) is a stress-responsive hormone that plays important roles in drought sensing and responses (Vargas et al., 2014). The CBF/DREB regulon is activated by the ABA-independent pathway (Saibo et al., 2009), and SlDREB3 expression in tomatoes influences several ABA-related processes by reducing ABA levels and responses, hence increasing photosynthesis (Upadhyay et al., 2017). Worchel et al. (2013) reported that the plant photosynthetic pathway affects arbuscular mycorrhizal (AM) fungal–plant symbiosis. Further, fungal community structure and diversity are affected by many environmental variables (Yang et al., 2017). Although DREB2s are known to contribute greatly to enhance drought and salinity tolerance in plants (Chen et al., 2009; Matsukura et al., 2010; Li et al., 2017), few studies have focused on the fungal communities in the soil of TG plants harboring DREBs (Yu et al., 2013).

Therefore, in the present study, we examined the effects of TG sugarcane harboring *Ea-DREB2B* on fungal communities in three soil compartments (rhizoplane, rhizosphere, and bulk soil). The aims of this study were as follows: (1) to determine the alteration in the diversity and composition of fungal communities in the rhizoplane, rhizosphere, and bulk soil around TG and non-TG wild-type (WT) plants; (2) to explore the associations between fungal community diversity and environmental factors (C, N, and P levels); and (3) to elucidate the complex interactions among fungi, plants, and the soil environment. These results can provide insights into the potential effects of using genetic modification to improve plant stress resistance in the context of a broader ecosystem, thereby offering guidance for burgeoning new genetically modified varieties of sugarcane.

## Materials and Methods

### Study Site

This study was conducted in the forage-growing area of Guangxi University, in Quli, Fusui, Chongzuo, China (between 107°31′ and 108°06′E, and 22°17′ and 22°57′N), in the summer of 2018. The average temperature during the study period was 21.3°C, and the total annual precipitation for the entire region was 1,050–1,300 mm (Supplementary Figure S1). The fields, which have been cultivated long term with sugarcane, had the following soil properties: lateritic red earth, pH of 5.15, 19.47 g/kg of organic matter, 0.84 g/kg of total N (TN), 2.98 g/kg of total P (TP), 7.11 g/kg of total K, 136 mg/kg of alkaline-hydrolyzed N, 83 mg/kg of available P, and 77.1 mg/kg of available K. We compared the properties and the fungal community diversity of the soil around the roots of the TG sugarcane variety GN18 with those of the WT variety FN95-1702. Xu et al. (2018) derived GN18 using FN95-1702 as the acceptor parent with the inducible promoter RD29A and a gene gun to achieve overexpression of *Ea-DREB2B* to improve drought resistance. Our experiment consisted of a random block design with six blocks containing both sugarcane varieties. In this experiment, random block design was adopted, with a total of six blocks, each with an area of 30 × 4.2 m. Each block contained two sugarcane varieties (three rows per plant). The distance between the two varieties was 2.1 m, and the distance between any two sugarcanes was 30 cm, with 46 sugarcane planted in each row (Zhao et al., 2020).

### Soil Sample Collection and Physicochemical Analysis

Sampling was conducted at the late jointing stage on November 18, 2018. Bulk soil was collected from five sampling sites between two lines of sugarcane with a standard soil corer each block using five-point sampling method. We excavated 12 sugarcane plants (six plants each variety) showing similar characteristics each block for extracting soil samples of rhizoplane and rhizosphere, and each replicate was mixed with soil collected from 12 sugarcane roots, which were excavated from two blocks through random selection in six blocks (Zhao et al., 2020). Rhizoplane soil sampled from the plant root surface was removed by sonication for 5 min (Edwards et al., 2015). Rhizosphere soil samples were separated by vortexing the roots for 20 s. Each composite soil sample was homogenized and stored at −80°C for less than 24 h before DNA extraction. We divided each soil sample into three technical replicates of 0.5 g each for physicochemical analysis. Total carbon (TC) (Batjes, 1996) and TP (Sommers and Nelson, 1972) contents were measured as previously described, and TN content was measured using the Kjeldahl method (Bremner and Tabatabai, 1972).

### DNA Extraction, Polymerase Chain Reaction, and Illumina Sequencing

Microbial DNA was extracted for each soil sample (3 replicates × 1 g) using the E.Z.N.A Soil DNA kit (Omega Bio-tek, Inc., Norcross, GA, United States) following the manufacturer’s instructions. The fungal internal transcribed spacer-2 (ITS-2) region was amplified from each sample using the primers ITS3F (5′-GCATCGATGAAGAACGCAGC-3′) and ITS4R (5′-TCCTCCGCTTATTGATATGC-3′) (Tj et al., 1990). Primers were synthesized by Invitrogen (Invitrogen, Carlsbad, CA, United States). PCRs, containing 25 μl of 2 × Premix Taq (Takara Biotechnology, Dalian Co., Ltd., China), 1 μl of each primer (10 mM), and 3 μl of DNA (20 ng/μl) template in a volume of 50 μl, were amplified by thermocycling: 5 min at 94°C for initialization; 30 cycles of 30-s denaturation at 94°C, 30 s annealing at 52°C, and 30- s extension at 72°C; followed by 10 min of final elongation at 72°C. The PCR instrument was BioRad S1000 (Bio-Rad Laboratory, CA, United States). The PCR was conducted using the S1000 Thermal Cycler (Bio-Rad) with initial denaturation for 5 min at 94°C, followed by 30 cycles at 94°C for 30 s, 52°C for 30 s, 72°C for 30 s, and 72°C for 10 min. The PCR products were sequenced by Magigene Technology (Guangzhou, China) using an Illumina HiSeq 2500 platform. Sequences analyses were performed by usearch software (V10^1^). Sequences with ≥ 97% similarity were assigned to the same OUT. Sequences were assigned to each sample based on their unique barcode and primer using Mothur software (V1.35.1^2^), after which the barcodes and primers were removed and got the effective Clean Tags. The ITS-2 sequences obtained in this study have been deposited in the National Center for Biotechnology Information Sequence Read Archive (SRA) database with accession number SRP257722.

### Statistical and Bioinformatics Analysis

Alpha diversity was estimated from the richness, Shannon diversity, and evenness of the fungal operational taxonomic units (fOTUs). Correlations between alpha diversity and soil properties were determined using the “corrplot” package (Wei et al., 2017) in R v. 3.6.3. Changes in the relative abundance of fungal communities in each compartment were evaluated using the “alluvial” and “ggplot” packages in R v. 3.6.3 (Wickham and Wickham, 2007). Distance-based redundancy analysis (dbRDA) was used to evaluate the relationships between soil characteristics and soil fOTUs. Mantel test was used to study the relationship between alpha diversity and environmental factors and between the enriched fOTUs and environmental factors. Mantel tests, principal coordinate analysis (PCoA), and dbRDA were performed using the “vegan” package in R v. 3.6.3 (Oksanen et al., 2013). All statistical analyses (analysis of variance and Tukey’s *post hoc* test) and Spearman’s rank correlations between phylum abundance and soil properties were performed using SPSS v. 22.0 (SPSS Inc., Chicago, IL, United States). The effect of soil compartments on fOTU abundance was investigated using edgeR (Robinson et al., 2010) on trimmed mean of M-values (TMM)-normalized data (Robinson and Oshlack, 2010) and visualized using ternary plots. TMM-normalized data were then used to calculate the Spearman rank correlations between OTUs to construct co-occurrence networks.

## Results

### Fungal Alpha Diversity and Beta Diversity

**FIGURE 1:**
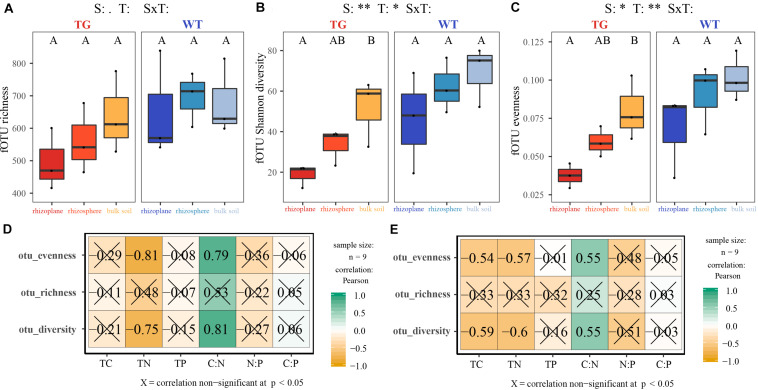
**(A–C)** Fungal alpha diversity indices represented by richness, Shannon diversity and evenness in each soil compartment and variety. **(D,E)** The correlation between indices of fungal alpha diversity in TG **(D)** and WT **(E)** and environmental factors using Pearson analysis. *0.01 < *P* value < 0.05; **P value < 0.01.

**FIGURE 2:**
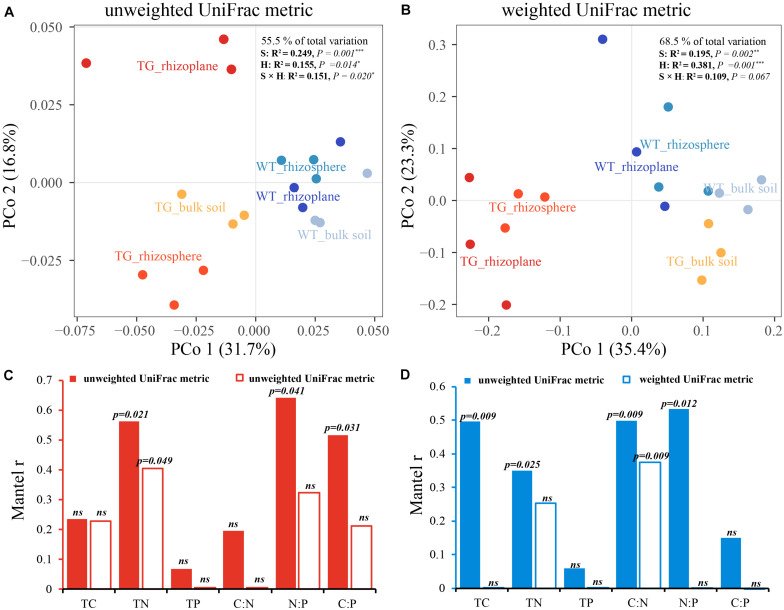
**(A,B)** Principal coordinate analyses (PCoAs) using unweighted UniFrac metric **(A)** and weighted UniFrac metric **(B)** indicate that the largest separation between fungal communities is spatial distribution of three areas (PCo1) and the second largest source of variation is cultivar (PCo2). **(C,D)** Correlation between environmental factors and phylogenetic membership and composition in two sugarcanes by using Mantel test.

The indices of fOTU richness, Shannon diversity, and evenness were used to represent fungal alpha diversity. These indices of TG sugarcane in all three soil compartments were lower than those of WT plants (Figures 1A–C). Additionally, the indices of fOTU Shannon diversity and evenness were different between rhizoplane and bulk soil in TG sugarcane, which was not observed in WT sugarcane. Pearson correlation analysis indicated that fungal alpha-diversity indices in two sugarcanes were both positively correlated with the C:N ratio but were negatively correlated with other environmental factors (Figures 1D,E). Besides, the TC and TN contents increased in TG root-associated areas (rhizoplane and rhizosphere) but decreased in the TG bulk soil area compared with those of WT (Supplementary Table S1). Phylogenetic analysis of fungal membership and composition was performed to evaluate fungal beta diversity using unweighted and weighted UniFrac distances, respectively (Figures 2A,B). The PCoA plots indicated good separation of weighted UniFrac distances for the soil fungal communities in different soil compartments (Figure 2B). Additionally, the fungal composition of the TG plant soil was remarkably different from that of WT soil based on weighted UniFrac metric. The mantel text indicated that the phylogenetic composition based on weighted UniFrac metric significantly contributed to the separation of fungal communities in both varieties. Further, a different relationship was observed between fungal structure based on weighted UniFrac metric and environmental factors in two sugarcanes. Fungal structure based on weighted UniFrac metric in TG showed no relationship with TC and C:N, while in WT sugarcane, fungal structure based on weighted UniFrac metric was significantly related with TC and C:N (Figures 2C,D).

### Fungal Community Composition and Abundance

**FIGURE 3:**
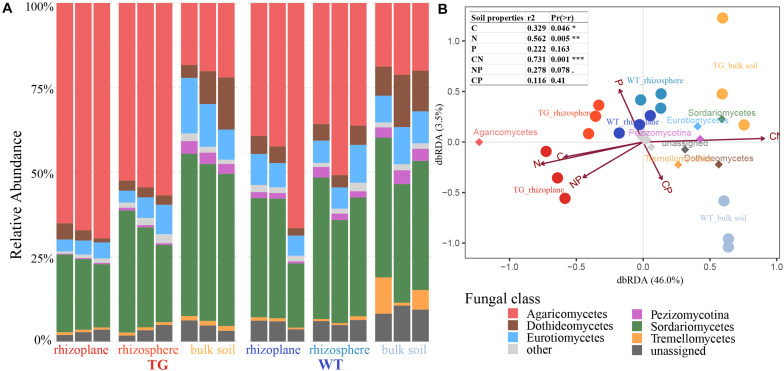
**(A)** Fungal relative abundance with class of each compartment of two sugarcanes, with three repetitions in each compartment. **(B)** Distance-based redundancy analysis of different zones, abundant classes, and six environmental factors (arrows) indicates the dominant communities and influential environmental factors.

There was a marked difference in fungal relative abundance at the class level between the TG and WT varieties. Compared with that with the WT plant, the relative abundance of Agaricomycetes was significantly higher, whereas the abundances of Dothideomycetes, Pezizomycotina, Eurotiomycetes, and Sordariomycetes were lower in root-related area of TG plants. In the bulk soil, the relative abundances of Eurotiomycetes and Sordariomycetes were higher for TG than for WT plants. And, the relative abundance of Tremellomycetes was significantly higher in the bulk soil of WT plants compared with that with of TG plants (Figure 3A). Based on dbRDA, the soil compartment and sugarcane variety explained 46.0 and 3.5% of the variation in fungal composition, respectively. The dominant fungal phyla across all soil samples were Agaricomycetes, Sordariomycetes, Eurotiomycetes, Pezizomycotina, Dothideomycetes, and Tremellomycetes. Apart from Agaricomycetes, which was found in the rhizoplane and rhizosphere of TG plants, Sordariomycetes, Eurotiomycetes, Pezizomycotina, Dothideomycetes, and Tremellomycetes were all present in the bulk soil of TG plants. Among the soil properties, C (*r*^2^ = 0.329, *P* = 0.046), N (*r*^2^ = 0.562, *P* = 0.005), and C:N (*r*^2^ = 0.731, *P* = 0.001) were significantly correlated with the distribution of fungal species (Figure 3B).

### Fungal Abundance Patterns in Transgenic and Wild-Type Plants

**FIGURE 4:**
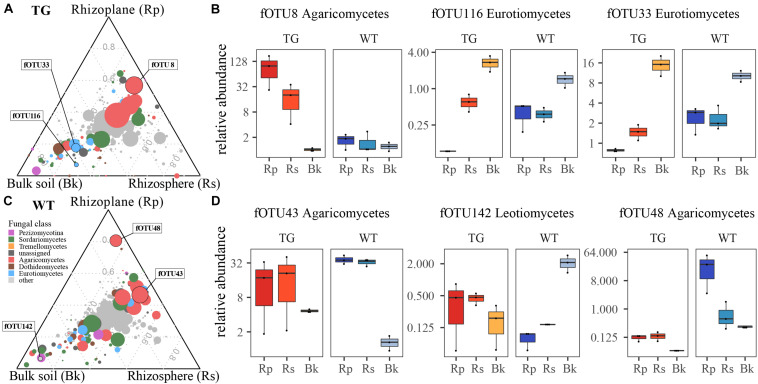
**(A,C)** Identifying fungal operational taxonomic units (fOTUs) that vary in abundance in response to different soil compartment in two sugarcanes. **(B,D)** The relative abundances of a few representatively responsive fOTUs in rhizoplane, rhizosphere, and bulk soil compartments, which are also indicated in the ternary plots.

We identified fOTUs that were differentially abundant among the soil compartments. Three fOTUs (fOTU8, fOTU116, and fOTU33) exhibited the largest differences in abundance for TG plants. For the WT plants, fOTU43, fOTU142, and fOTU48 were the most differentially abundant OTUs. Agaricomycetes (fOTU8, fOTU43, and fOTU48) were enriched in the root-related area of both TG and WT plants. Eurotiomycetes (fOTU116 and fOTU33) was enriched in the bulk soil of TG plants, whereas the abundant fungal class Leotiomycetes (fOTU142) was found to be enriched in the bulk soil of WT plants (Figure 4).

**FIGURE 5:**
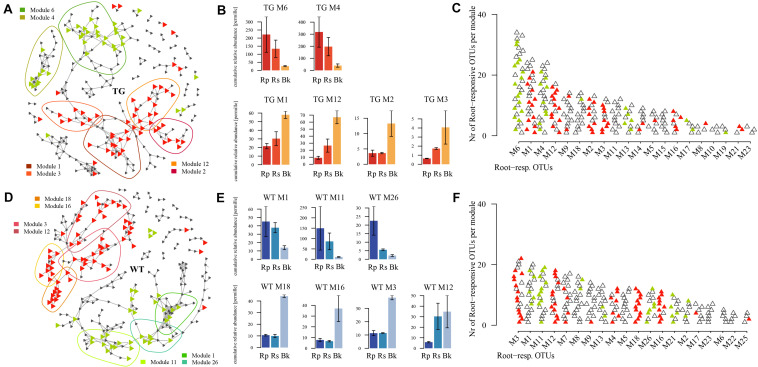
**(A,D)** Co-occurrence networks visualize the significant positive pairwise correlations [*r* > 0.7, *P* < 0.001; indicated by links between operational taxonomic units (OTUs)] between fOTUs (triangles) in transgenic (TG) **(A)** and wild type (WT) **(D)**. The green triangles within the green circle indicate the root-enriched (rhizoplane-enriched and rhizosphere-enriched) fOTUs, and the red triangles in the red circle indicate the bulk soil-enriched fOTUs. **(B,E)** Cumulative relative abundance of all fOTUs in the responsive modules in root-enriched and bulk soil-enriched areas. The cumulative relative abundance indicates the overall response of the fungi in the responsive modules. **(C,F)** Top 20 most populated modules, ranked by decreasing numbers of fOTUs with root-enriched and bulk soil-enriched fOTUs being colored in green and red, respectively.

We employed co-occurrence network analysis to identify groups of fungi with similar abundances according to the soil compartment. The groups were separated into six and seven modules (for TG and WT plants, respectively) based on similarities in abundance. There was marked separation of fungal groups between the root-related area (rhizoplane and rhizosphere) and bulk soil of TG plants (Figure 5A). Modules M6 (representing Sordariomycetes) and M4 (representing Agaricomycetes) comprised a high proportion of the fOTUs (Figure 5C) and were specifically abundant in the root-enriched area of TG plants (Figure 5B). Modules M1, M12, M2, and M3, which comprised a relatively high proportion of fOTUs (Figure 5C), were enriched in the bulk soil of TG plants (Figure 5B). There was similar separation of fOTUs between the root-related area and bulk soil of WT plants (Figure 5D). Modules TM1, TM11, and TM26 were mainly present in the root-enriched areas of WT plants (Figure 5E), whereas modules TM18, TM16, TM3, and TM12, which comprised fOTUs that were more abundant (Figure 5F), were enriched in the bulk soil of WT plants. Further, a set of fOTUs in Chytridiomycetes and Ustilaginomycetes were detected in the root-enriched area of TG plants, with no presence in the WT-enriched area. The disputed class Zygomycota (Zygomycota_cls_Incertae_sedis) was found only in module M3 in the bulk soil-enriched area of TG plants, whereas Tremellomycetes were found only in module TM18 in the bulk soil-enriched area of WT plants (Supplementary Figure S2). Apart from these species, other fungal (Eurotiomycetes, Sordariomycetes, Pezizomycotina_cls_Incertae_sedis, Dothideomycetes Agaricomycetes, and unassigned) groups were present in both TG and WT sugarcanes (Supplementary Figure S2). The relative abundances of Agaricomycetes, Sordariomycetes, and Dothideomycetes all declined in the root-enriched area but increased in the bulk soil-enriched area of TG sugarcane compared with those of WT plants. The relative abundance of Eurotiomycetes and Pezizomycotina_cls_Incertae_sedis declined in root- and bulk soil-enriched areas of TG sugarcane compared with that of WT plants (Supplementary Figure S3).

### Correlations Between Fungal Operational Taxonomic Units and Soil Properties and Their Functional Roles

**FIGURE 6:**
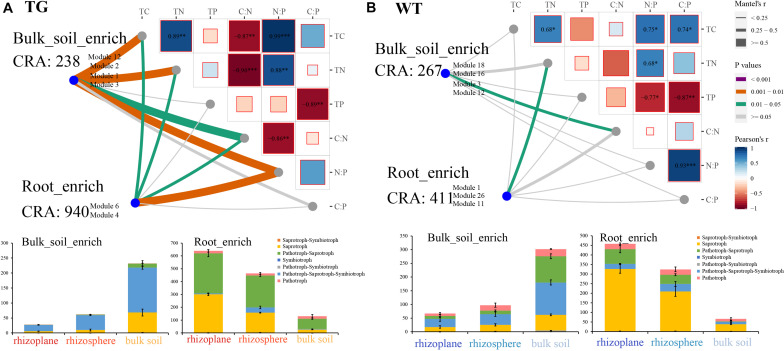
**(A)** Correlation between fungal operational taxonomic units (fOTUs) represented by cumulative relative abundance (CRA) in root-enriched modules (M6 and M4) and bulk soil-enriched modules (M1, M12, M2, and M3) and environmental factors in transgenic (TG) plant using Mantel test; fungal trophic modes of fOTUs enriched in TG root and bulk soil areas. **(B)** Correlation between fOTUs in root-enriched modules (M1, M11, and M26) and bulk soil-enriched modules (M16, M3, and M12) and environmental factors in WT plant using Mantel test; fungal trophic modes of fOTUs enriched in WT root and bulk soil areas.

We used Mantel testing to analyze the relationship between fOTUs in root-enriched and bulk soil-enriched areas and environmental factors. Root-enriched fOTUs indicate the OTUs, as shown in Figure 4 (M6 and M4 in TG; and M1, M11, and M26 in WT), that were relatively enriched in the rhizoplane and rhizosphere. Similarly, bulk soil-enriched fOTUs indicate the OTUs (M1, M12, M2, and M3 in TG, and M18, M16, M3, and M12 in WT) that were relatively enriched in bulk soil. The relative abundance of TG root-enriched fOTUs of 940 was far more than that of WT root-enriched fOTUs of 411. The internal correlations among the environmental factors were stronger in the soil of the TG plants than in that of WT plants. The fOTUs in the TG plant soil compartments were significantly correlated with C, N, and C:N and N:P ratios. However, the bulk soil-enriched area fOTUs were more highly correlated with environmental factors than were root-enriched fOTUs in TG plants (Figure 6A). The correlation between fOTUs and environmental factors was weaker in the soil of WT plants than in that of TG plants. The fOTUs in the WT root-enriched area were related only to TN, and those in the WT root-enriched area were related only to the C:N ratio (Figure 6B). The internal relationships among environmental factors were significantly stronger in TG than in WT plants. Functional annotation of the OTUs predicted seven trophic modes, including saprotroph–symbiotroph, saprotroph, pathotroph–saprotroph, symbiotroph, pathotroph–symbiotroph, pathotroph–saprotroph–symbiotroph, and pathotroph. Among the fOTUs in both the TG root-enriched area and WT root-enriched area, most fOTUs were assigned to the saprotroph and pathotroph–saprotroph function (Figures 6A,B). However, the quantity of TG root-enriched fOTUs was larger than that of WT. Most fOTUs in the TG plant bulk soil-enriched area were categorized as pathotroph–saprotroph–symbiotroph (Figure 6A).

## Discussion

### Variation in Fungal Diversity in the Soil of Transgenic Plants

In the present study, differences of the fungal diversity have been observed in all three compartments of TG and WT sugarcanes under the same planting conditions. However, Romão-Dumaresq et al. (2016) have indicated that the plant genotype (TG and non-TG) does not significantly change the fungal diversity of sugarcane in the rhizosphere, which is the opposite of our results. As previous studies showed, fungal diversity was affected in lettuce and *Arabidopsis thaliana* due to plant genotype (Hunter et al., 2015; Urbina et al., 2018), while no significant differences of fungal diversity between the WT and TG lines were found in potato and rice (Milling et al., 2005; Sohn et al., 2016). These conflicting results arise from different functional genes in plants. *Ea-DREB2B* belongs to the DREB subfamily, the members of which are capable of regulating drought response via ABA-dependent as well as ABA-independent pathways (Vargas et al., 2014). Thus, altered ABA pathways in TG plants affect the pattern of TG sugarcane exudates to some extent due to the overexpression of *Ea-DREB2B*. Accumulating evidence suggests that plant-produced metabolites can significantly shape the microbiome composition and activity (Dunfield and Germida, 2004). Root exudation was previously shown to enhance sugarcane rhizosphere fungal activity, which can in turn affect plant growth and fitness via hormone production or the mineralization of available nutrients (Lian et al., 2018). In our results, the roots structure of TG sugarcane was significantly more developed than that of WT (Supplementary Figure S4), and C and N contents in TG root-associated compartments increased compared with those of WT (Supplementary Table S1), suggesting a more nutritious living environment for TG fungal species and plant roots. Interestingly, unlike the relationship trend observed between indices representing alpha diversity in both TG and WT and environmental factors, the phylogenetic composition based on weighted UniFrac metric representing beta diversity of TG fungal species was not related to C:N, which differed from that of species with WT. It has been reported that fungi can endure environmental changes that are less well tolerated by other organisms, indicating a more stable structure of fungal species than that of other microorganisms (de Vries et al., 2018). Indeed, comparing the present results with the results of our previous study (Zhao et al., 2020), the structure of fungal species around plant roots was less affected by changes in soil environment compared with that of bacteria in TG plants. Overall, our results reveal that fungal diversity is affected by changes in soil living environment that primarily result from plant genotype.

### Variation in Fungal Community Composition Between Sugarcane Varieties

There was greater variation in fungal composition between the root-associated areas and bulk soil with TG plants than with WT plants. Fungal communities are recruited from the soil in a manner consistent with the two-step selection of bacterial communities: first from the bulk soil to the rhizosphere and then from the rhizosphere to the endosphere (Bulgarelli et al., 2013). We found clear alterations in all three soil compartments with TG sugarcane compared with those with WT plants (Figure 3A), suggesting that the soil fungal communities migrated under the influence of genotypes. Taking the two-step recruitment of fungal communities into consideration, we speculate that the soil fungal community of TG sugarcane migrated from outside to the inside, from the bulk soil to the rhizosphere, from the rhizosphere to the rhizoplane, and finally into the root. The host plant greatly affects the fungal diversity and composition in the soil around the roots, and certain fungal species are particularly affected by the host plants (Urbina et al., 2018). We found a markedly higher relative abundance of Agaricomycetes in the rhizoplane and rhizosphere of TG sugarcane compared with that of WT plants, but a higher relative abundance of Sordariomycetes and Eurotiomycetes was observed in the bulk soil of TG plants compared with that of WT plants (Figure 3A), suggesting fungal colonization, particularly by certain fungal species. Agaricomycetes accounts for the major mycorrhizal taxa, which can form a mutualistic symbiotic relationship with the roots of plants to enhance the uptake of immobile nutrients from the soil (Douds and Millner, 1999). Inoculation with *Funneliformis mosseae* (an AM fungus) on the roots of trifoliate orange enhances the relative abundance of Sordariomycetes and has been widely reported to improve soil health (He et al., 2019). Sordariomycetes comprises one of the major responders to drought stress (Meisner et al., 2018). Members of Ascomycota, the phylum containing Sordariomycetes and Eurotiomycetes, can tolerate stressful conditions such as low nutrient availability, enabling them to achieve more efficient resource use in challenging environments (Li et al., 2020). Notably, TC, TN, and the C:N ratio were the most important factors explaining fungal community structure (Figure 3B), indicating that changes in fungal composition are caused by the effects of fungi altering the physiochemical environment. Fungal community colonization depends on environmental conditions when the resource becomes available for colonization; that is, when the habitat is altered, most fungal communities are usually replaced by other species that are more combative or better able to tolerate conditions within the resource by virtue of certain modifications in their structure or physiology (Boddy and Hiscox, 2017).

### Enhanced Relationship Between Transgenic-Enriched Fungal Operational Taxonomic Units and Environmental Factors

Analysis of the network co-occurrence patterns of two sugarcane types showed the same trend in our study: certain fOTUs of both varieties were enriched in the root and bulk soil areas (Figure 5). However, a significantly closer relationship was observed between isolated fOTUs in two enriched areas and environmental factors in TG plants than in WT plants (Figure 6). These phenomena indicate that, to a certain degree, the structure or function of fungal species around TG roots changed compared with that around WT. As a multitudinous class of soil microorganisms, fungi comprise multiple functional groups, including decomposers, mutualists, and pathogens (Bardgett and Wardle, 2010). Saprotrophs, a group of fungi making up 50–80% of the overall fungal community (Schmidt et al., 2019), are principal decomposers in terrestrial habitats. In our study, more saprotrophs were enriched in TG root areas than in WT root areas, indicating the increased proportion of saprotrophs and the altered fungal-rhizosphere niche. As a previous study showed, soil environmental changes may shift the original niches of the three fungal guilds (pathotroph, saprotroph, and symbiotroph) in root-associated areas, potentially influencing decomposition within that ecosystem (Bödeker et al., 2016). Furthermore, it has been reported that the rate of decomposition is intimately related to the C:N ratio (Dighton, 2007), and a reduced decomposition rate has been shown with higher C:N than with lower ratios, which is consistent with our results; we observed lower C:N in TG root-related areas than in that of WT with increased saprotrophs (Supplementary Table S1). It has also been reported that, compared with ectomycorrhizal fungi (symbiotrophic fungi), saprotrophs even play a more important role in the production of labile C-targeting hydrolase enzymes (Talbot et al., 2013). Additionally, fungal species that colonize bulk soil and that have no interaction with plant roots seem to depend on nutrients inherent in the soil to acquire nutrition, which may explain the close relationship between bulk soil-enriched fOTUs and environmental factors. Thus, enhanced relationship between TG root-related fOTUs and environmental factors and altered fungal niches with more saprotrophs around the TG roots develop the beneficial function of fungi and have a positive impact on the environment by facilitating plant growth and development, thereby further enhancing plant resistance to drought stress.

## Conclusion

Genetic regulation is considered a crucial mechanism by which plants respond to drought stress. In this study, we applied several analytical approaches to investigate the impacts of TG *Ea-DREB2B*-carrying sugarcane plants on the fungal communities in the soil. Our study revealed that alterations in fungal communities by TG sugarcane are closely related to environmental factors. Our study also revealed that the apparent migration of fungal communities in the soil compartments of TG sugarcane corresponds to changes in their living environment. Additionally, an enhanced relationship between fOTUs and environmental factors and more abundant saprotrophs were observed in TG root-enriched areas, suggesting strengthened plant–fungal interactions in TG-enriched areas. Furthermore, judging from the results of microbial diversity and network analyses in the present study and our previous study, the structure of fungal species is more stable than that of bacterial communities with changing living environment due to different plant genotype (Zhao et al., 2020). Although we did not find a direct relationship between the enhanced drought response of TG sugarcane and altered fungal communities, the increase in specific beneficial fungi in TG soil compartments and the enhanced relationship between TG-enriched fOTUs and environmental factors contribute substantially to improving the drought response capability of TG sugarcane. However, it should be recognized that soil fungal communities are affected by many other natural sources of variation. Therefore, more comprehensive studies on the effects of TG plants on fungal communities in the soil, considering other potentially important factors, are required.

## Data Availability Statement

The datasets presented in this study can be found in online repositories. The names of the repository/repositories and accession number(s) can be found below: https://www.ncbi.nlm.nih.gov/, SRP257722.

## Author Contributions

XZ, ZW, QL, and SX contributed to design of the experiments, data analysis, and manuscript writing. XZ, QL, SX, and HY contributed to experimentation. HY and MZ contributed to data interpretation. All authors contributed to the article and approved the submitted version.

## Conflict of Interest

The authors declare that the research was conducted in the absence of any commercial or financial relationships that could be construed as a potential conflict of interest.

## Footnotes

1 http://www.drive5.com/usearch/

2 http://www.mothur.org
