# Supplementary figures and images for: Response of Soil Fungal Community to Drought-Resistant Ea-DREB2B Transgenic Sugarcane
Source: Front Microbiol. 2020 Sep 18;11:562775. doi: 10.3389/fmicb.2020.562775 (PMC7530946; doi:10.3389/fmicb.2020.562775)

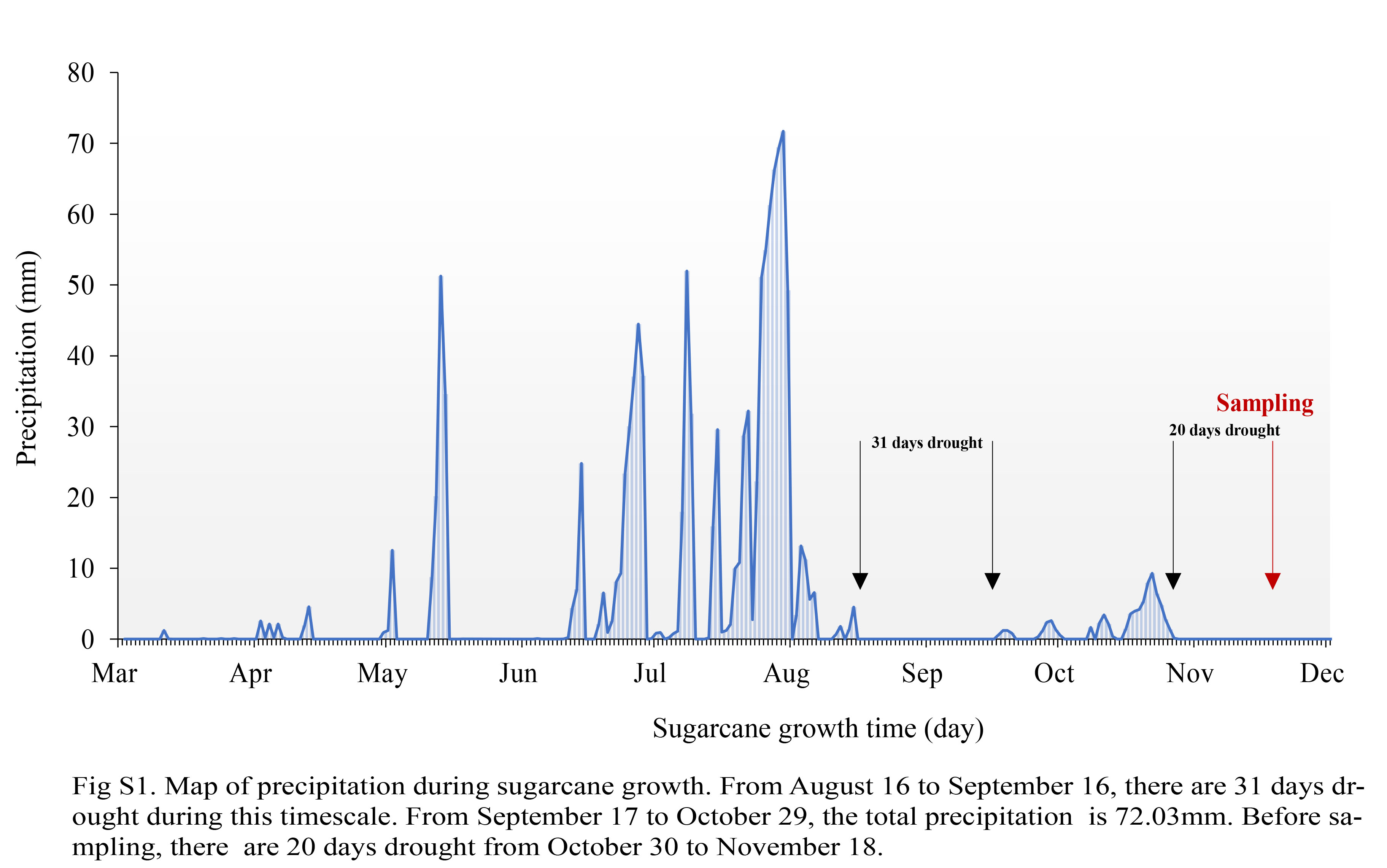

Supplement: Supplementary file 1 [file Image_1.JPEG]

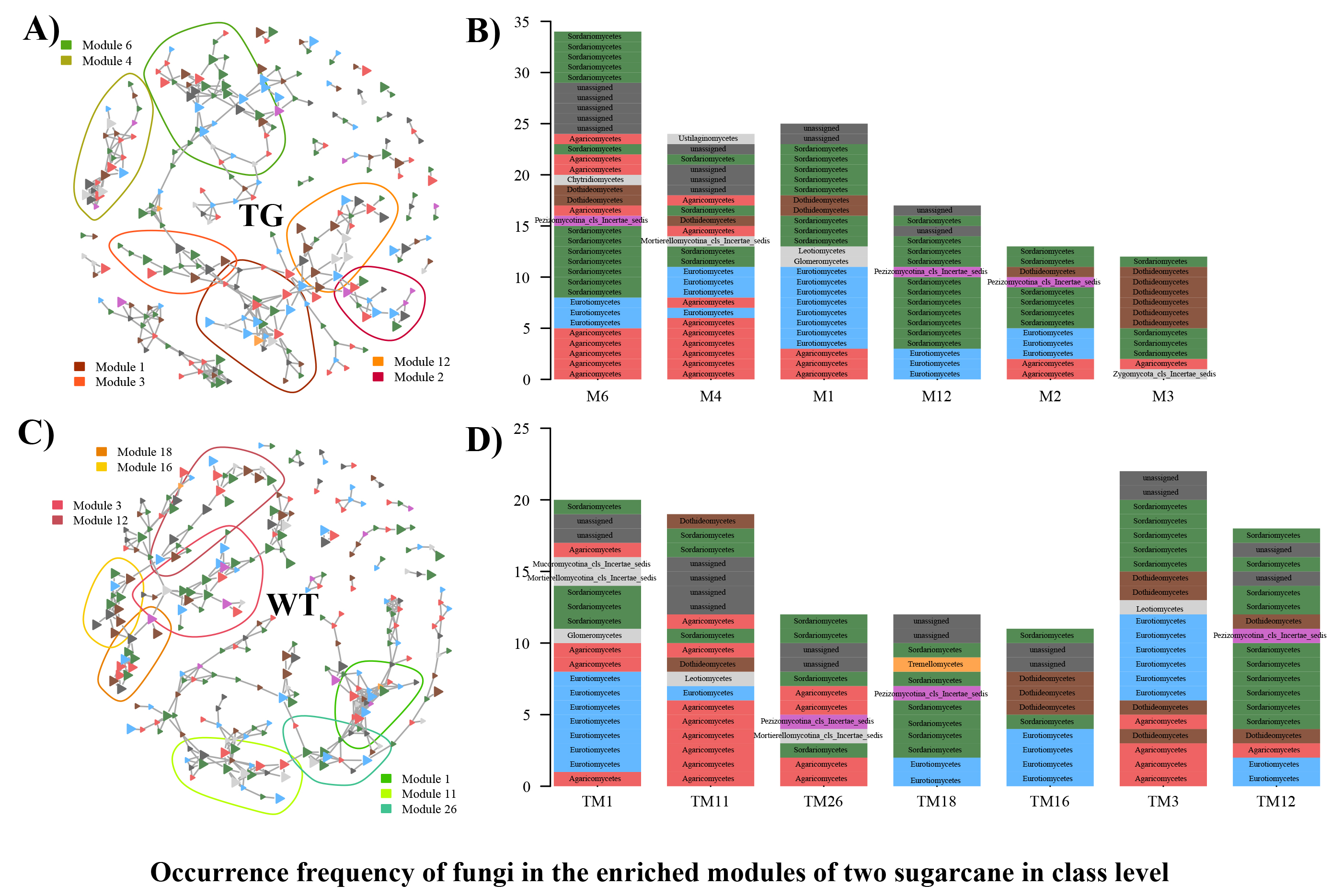

Supplement: Supplementary file 2 [file Image_2.JPEG]

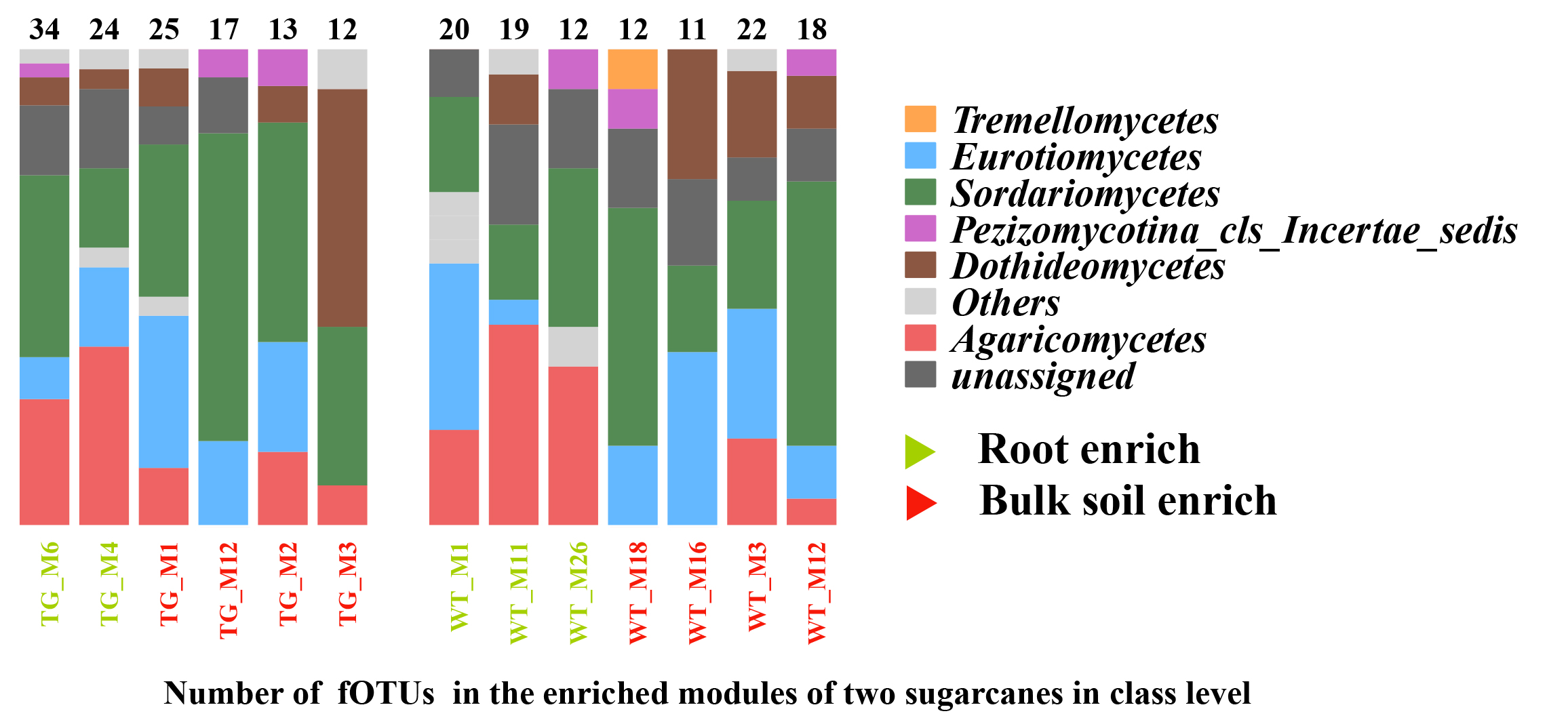

Supplement: Supplementary file 3 [file Image_3.JPEG]

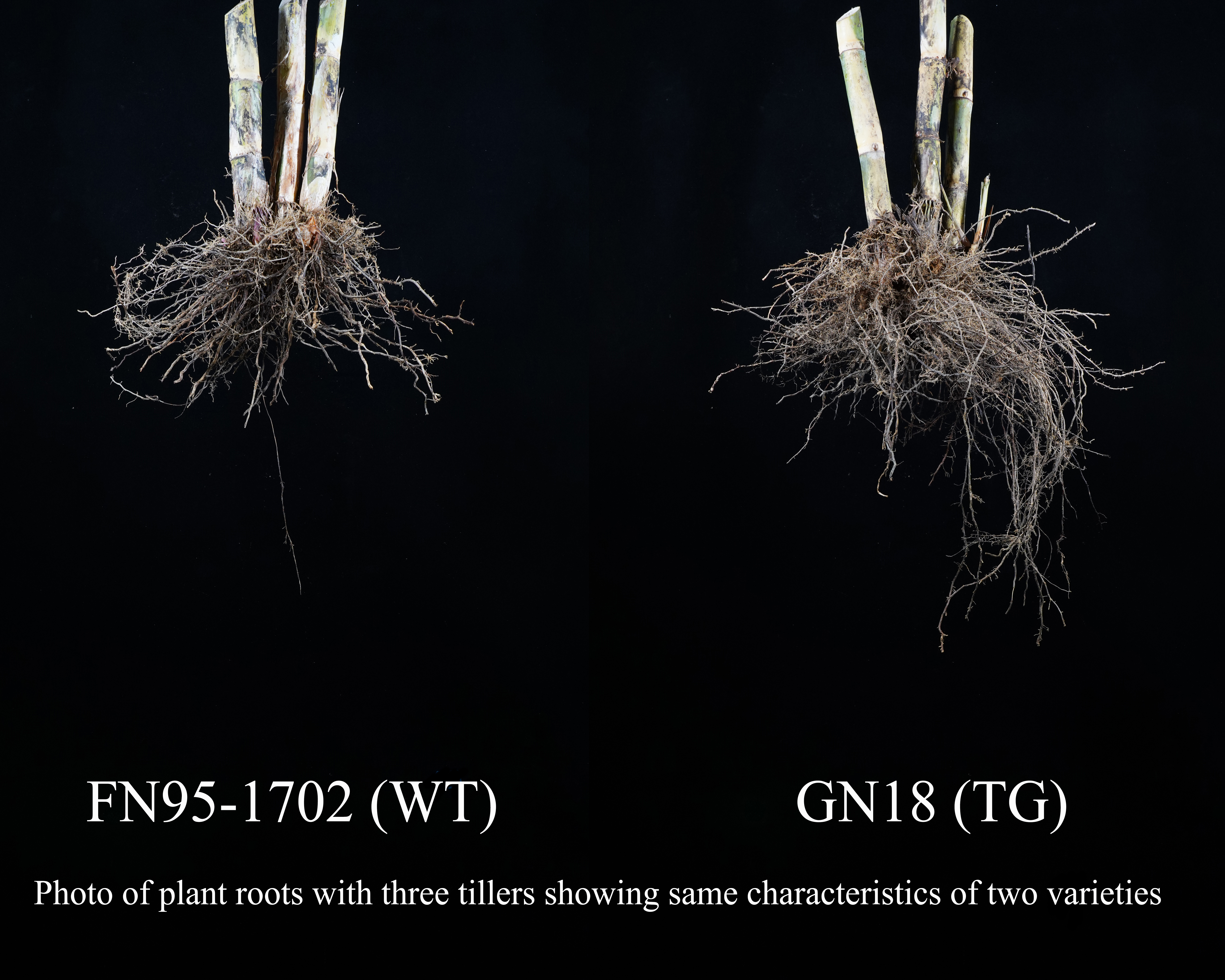

Supplement: Supplementary file 4 [file Image_4.JPEG]
