# Supplementary material for: Response of Soil Fungal Community to Drought-Resistant Ea-DREB2B Transgenic Sugarcane
Source: Front Microbiol. 2020 Sep 18;11:562775. doi: 10.3389/fmicb.2020.562775 (PMC7530946; doi:10.3389/fmicb.2020.562775)
Supplement: Supplementary file 5 [file Table_1.docx]

| Table 1  Soil chemical properties and the ratio between them according to different soil compartments | | | | | | | | | | | | | |
| --- | --- | --- | --- | --- | --- | --- | --- | --- | --- | --- | --- | --- | --- |
| **Line** | **soil compartment** | **TC(g·kg^-1^)** | | **TN(g·kg^-1^)** | | **TP(mg·kg^1^)** | | **TC/TN** | | **TN/TP** | | **TC/TP** | |
| **WT** | **Rhizoplane** | 14.03±2.62ab |  | 1.21±0.20a |  | 4.86±1.59a |  | 11.51±0.58b |  | 0.28±0.15b |  | 3.26±1.73a |  |
|  | **Rhizosphere** | 11.43±1.88ab |  | 0.88±0.02a |  | 10.82±0.66c |  | 12.95±2.37bc |  | 0.08±0.00a |  | 1.07±0.24a |  |
|  | **Bulk soil** | 11.88±1.95ab |  | 0.74±0.02a |  | 3.89±0.69a |  | 15.95±2.29c |  | 0.20±0.04ab |  | 3.18±1.04a |  |
| **TG** | **Rhizoplane** | 15.75±1.80b |  | 2.97±0.56b |  | 10.42±0.15bc |  | 5.35±0.42a |  | 0.29±0.06b |  | 1.51±0.19a |  |
|  | **Rhizosphere** | 13.52±0.58ab |  | 1.21±0.08a |  | 5.77±0.65ab |  | 11.22±0.25b |  | 0.21±0.04ab |  | 2.37±0.37a |  |
|  | **Bulk soil** | 9.73±0.91a |  | 0.67±0.05a |  | 10.45±3.75bc |  | 14.48±1.42bc |  | 0.07±0.02a |  | 1.04±0.44a |  |
| **Line** | | 0.518 |  | <0.001*** |  | 0.014* |  | <0.001*** |  | 0.938 |  | 0.057 | . |
| **Soil compartment** | | 0.006** |  | <0.001*** |  | 0.542 |  | <0.001*** |  | 0.005** |  | 0.432 |  |
| **Line ×Soil compartment** | | 0.110 |  | <0.001*** |  | <0.001*** |  | 0.032* |  | 0.026* |  | 0.009** |  |

a TC = total carbon; TN = total nitrogen; TP = total phosphate;

b Different letters indicate significant differences (ANOVA, P <0.05, Turkey’s HSD post-hoc analysis) among root compartment.

c ^*^0.01 < P value< 0.05; ^**^P value < 0.01; ^***^P value < 0.001.
